# Osteochondrolipoma of the foot treated by surgical excision: a case report and literature review

**DOI:** 10.1186/s12891-024-07308-1

**Published:** 2024-04-09

**Authors:** Fawzi Aljassir, Musab Alageel, Malak N. AlShebel, Abdulaziz M. Alsudairi, Ahmed Hashim, Ibrahim Alshaygy

**Affiliations:** 1 Department of Orthopedics, College of Medicine, King Saud University Medical City, Riyadh, Saudi Arabia; 2 College of Medicine, King Saud bin Abdulaziz University for Health Sciences, Riyadh, Saudi Arabia; 3 Bone and Joint Hospital, Dr Sulaiman Al Habib, Riyadh, Saudi Arabia; 4 College of Medicine, Alfaisal University, Riyadh, Saudi Arabia

**Keywords:** Lipoma, Osteochondrolipoma, Total excision

## Abstract

**Background:**

Osteochondromas, classified as a new benign subtype of lipomas and characterised by chondroid and osseous differentiation, are rare lesions that have been infrequently reported in previous literature. The maxillofacial region was reported as the most frequent localization, with infrequent occurrence in the lower limb. This paper represents the first documented case report of osteochondrolipoma in the foot.

**Case presentation:**

A 51-year-old male patient presented with a chief complaint of right foot pain at the plantar aspect, accompanied by the observation of swelling between the first and the second metatarsal shafts. His complaint of pain and swelling started 10 and 4 years prior, respectively. Since their onset, both symptoms have progressed in nature. Imaging revealved a large mass exhibiting a nonhomogenous composition of fibrous tissue and bony structures. Surgical intervention through total excision was indicated.

**Conclusion:**

Osteochodrolipoma is a benign lesion that can affect the foot leading to decreased functionality of the foot due to the pain and swelling. Surgical excision is the recommended approach for this lesion, providing both symptomatic relief and confirmation of the diagnosis through histopathological examination.

## Introduction

Lipomas have been reported to be the most prevalent benign soft tissue neoplasms, which are further classified into superficial and deep, depending on their location [1]. Differentiation into different mesenchymal elements, including fibrous tissues, blood vessels, or muscle, has been documented. However, differentiation into bone or cartilage has a low predilection and is often associated with parosteal localization [2]. Osteochondromas, classified as a new benign subtype of lipomas and characterized by chondroid and osseous differentiation, are rare lesions that have been infrequently reported in previous literature. The first case, observed by Soeder et al., presented with a 70-year-old male patient who underwent MRI of the left thigh, revealing the presence of nonhomogenous fibrinous tissue and bony structures. Furthermore, this isolated lesion was found to be independent of the neurovascular bundle and not attached to the bone. Histopathological examination demonstrated an encapsulated lesion with a smooth surface composed of vascularized fibrous capsule. Within the capsule, yellowish adipose tissue was observed, along with a notable presence of cartilaginous and bony components. Microscopic evaluation revealed a significant number of osteocytes, accompanied by a small rim of osteoblasts indicative of woven bone formation. Adjacent to the woven bone, cartilaginous tissue was also identified [3]. Other regions that have been reported in the literature include the forearm, ischial region, mandible, axilla, scapular region, popliteal fossa, and chest wall. Reports of this condition in the lower half of the body is relatively uncommon. All the reported cases treated this lesion with surgical excision, confirmed their diagnosis with histopathology, and reported no recurrence. Additionally, the patients were able to regain full functionality of the affected organ or limb [4–8].

This study provides a unique case of osteochondrolipoma, specifically located in the foot. This localization further adds to the rarity of this condition, as it has not been previously observed in such anatomical region. To the best of our knowledge, this represents the first documented case report of osteochondrolipoma in the foot. By highlighting this novel occurrence, our research expands on the current understanding of osteochondrolipoma and its diverse anatomical presentations.

## Case presentation

A 51-year-old male patient was presented to the orthopedic clinic at King Saud University Medical City on July 11, 2023. He presented with a chief complaint of right foot pain at the plantar aspect, accompanied by the observation of swelling between the first and the second metatarsal shafts. His complaint of pain and swelling started 10 and 4 years prior, respectively. Since their onset, both symptoms have progressed in nature. Prior to his presentation, the patient had been surgically and medically free, and able to walk and undergo regular daily activities with no limitations. Throughout the years, he had been taking non-steroidal anti-inflammatory drugs (NSAIDs), including meloxicam, for pain relief. A year prior to his presentation, the pain started impeding daily activities, and he found no further relief with the use of analgesia. The patient denied prior instances of swelling in other areas in his body. Additionally, he denied any history of trauma, or constitutional symptoms such as fever, fatigue, night sweats, weight loss or loss of appetite. Furthermore, the patient denied any history of smoking.

**Fig. 1:**
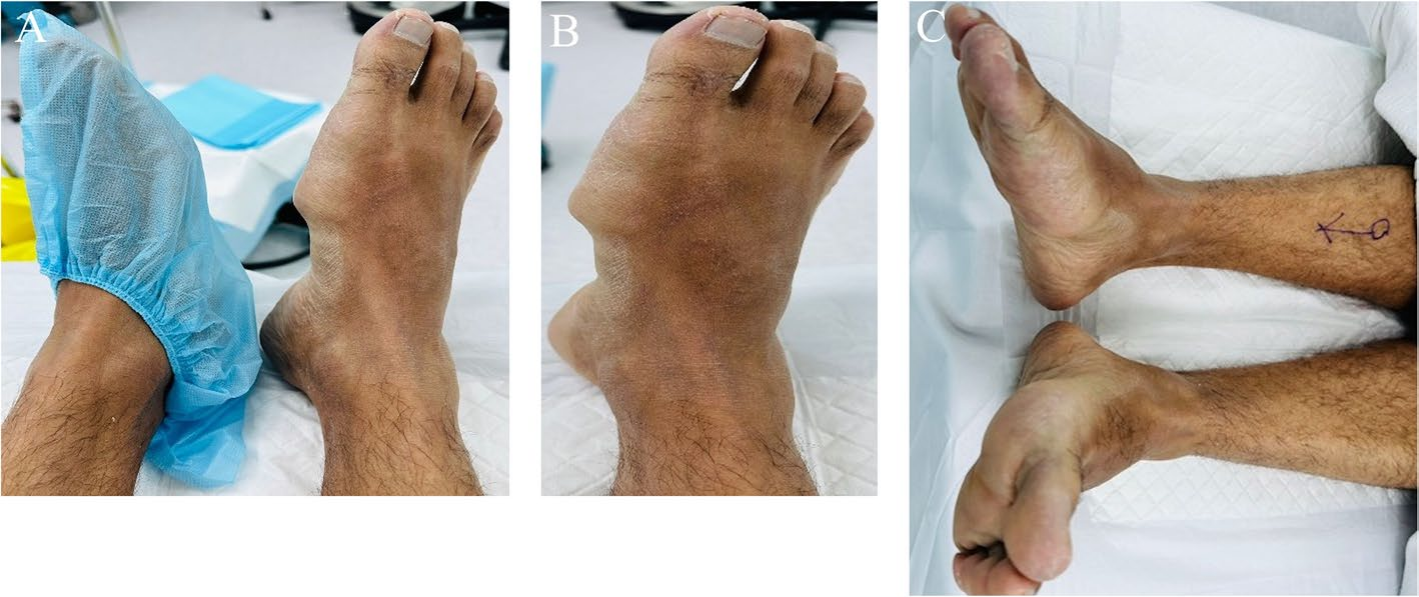
**A**.** B**.** C**.Clinical examination of the right foot

 Physical examination of his right foot revealed obvious swelling between the 1st and 2nd metatarsal shafts, extending to plantar aspect of the foot and the medial aspect of the first metatarsal shaft (Fig. 1). The swelling was 6*3 Cm in size, displaying an irregular shape accompanied by erythema. It was nonmobile, firm in its consistency, tender, and hot.

**Fig. 2:**
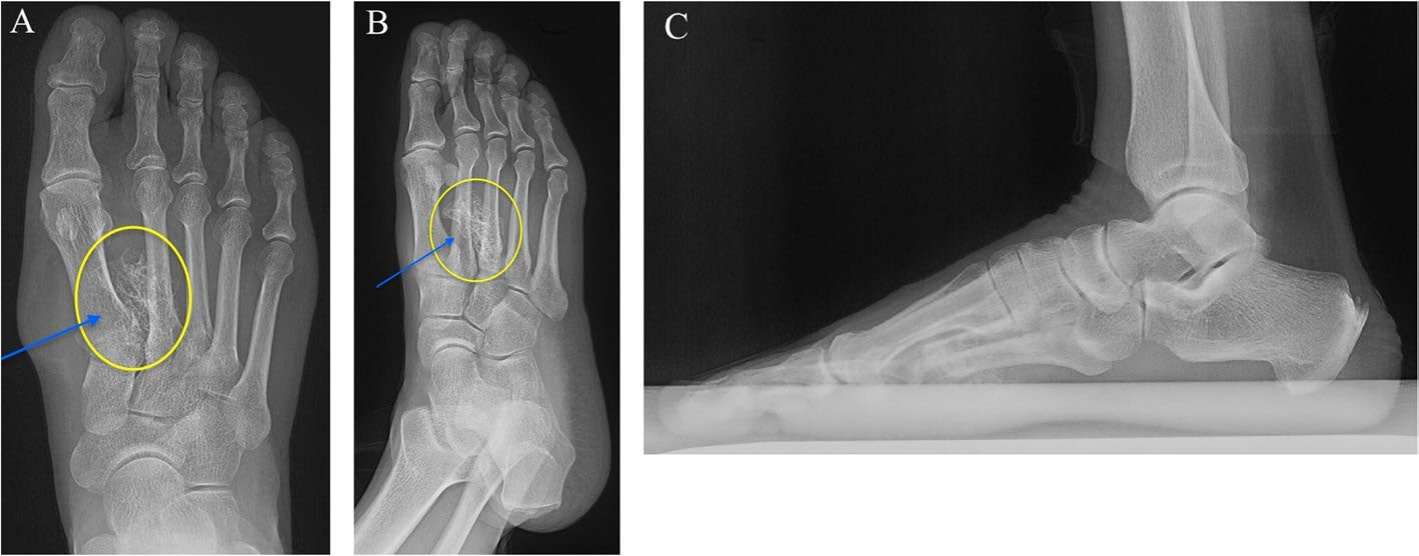
Preoperative radiographs of the right foot showing a 4*3 calcified lesion between the first and third metatarsal shafts with no bony involvement. **A** Anteroposterior (AP) view **B** Oblique view. **C** Lateral view

 The patient demonstrated normal plantar flexion and dorsiflexion of the ankle joint with no pain, as well as pain-free and normal eversion and inversion of the subtalar joint. However, due to pain in the first and second phalanges of the first and second rays, there was limited range of motion in that area. He was neurovascularly intact and exhibited an antalgic gait while mobilizing. Plain radiographs demonstrated soft tissue swelling with a medial surrounding of bone density (Fig. 2).

**Fig. 3:**
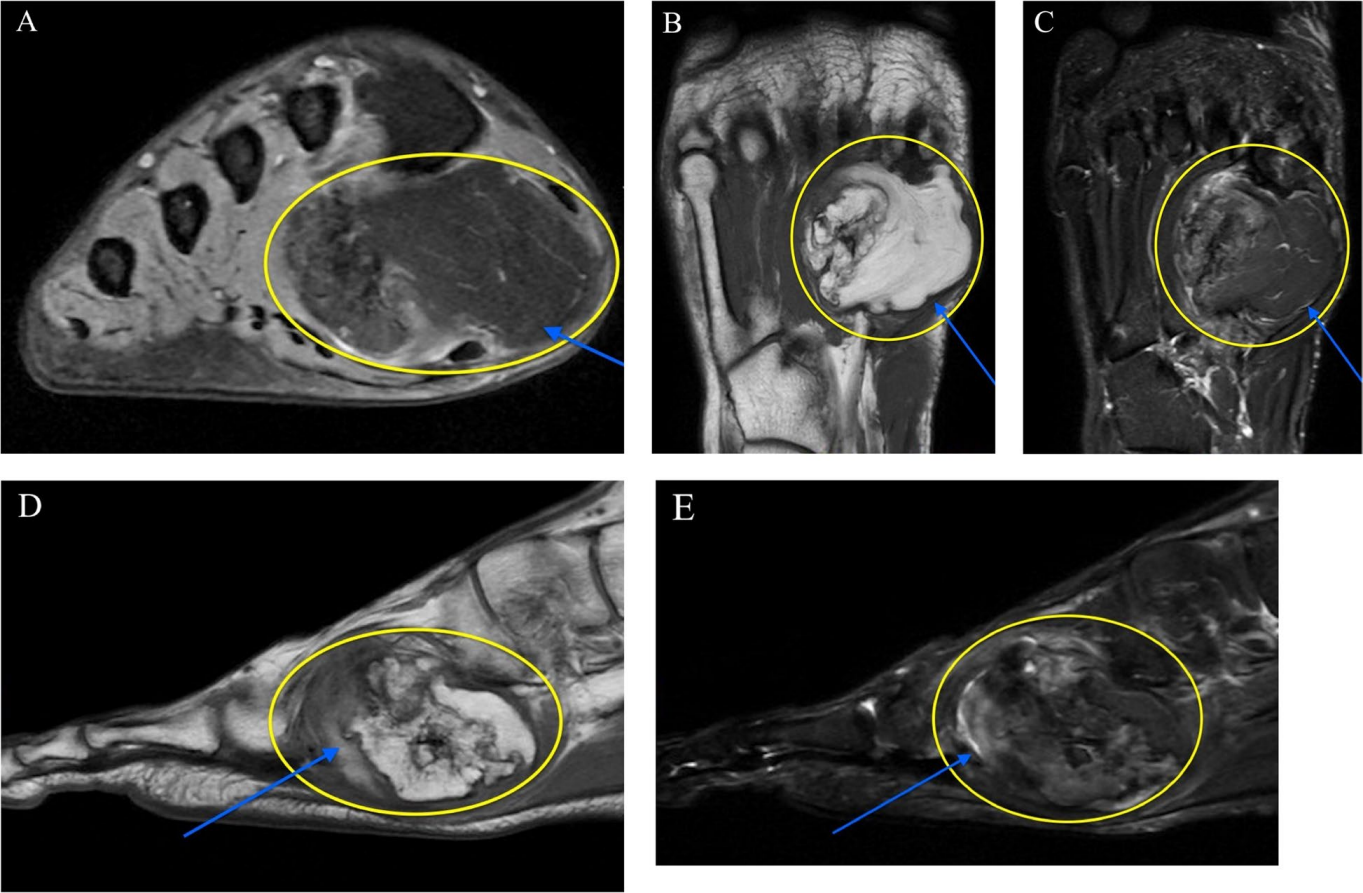
Preoperative MRI of the right foot showing a nonhomogeneous mass. **A **Axial T2-weighted. **B **Coronal T1-weighted. **C** Coronal T2-weighted **D **Sagittal T1-weighted. **E** Sagittal T2-weighted

 Further investigation was deemed necessary, prompting the indication of magnetic resonance imaging (MRI). MRI revealed a large mass between the first and third metatarsal shafts, extending to the plantar aspect of the foot. The mass exhibited a nonhomogenous composition of fibrous tissue and bony structures, and surgical intervention was indicated (Fig. 3).

**Fig. 4:**
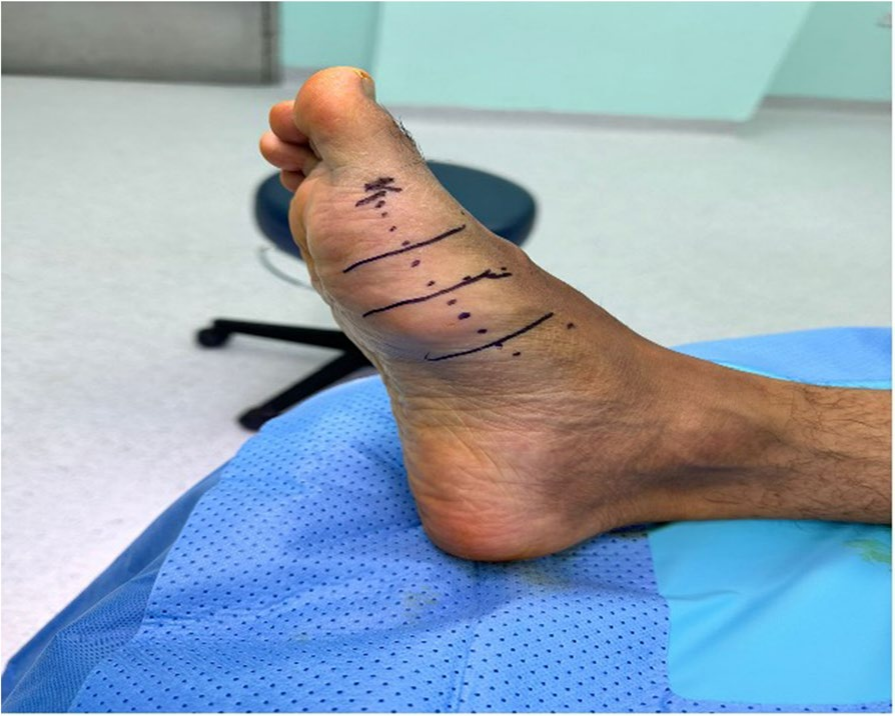
Right foot of the patient with medial incision markings

**Fig. 5:**
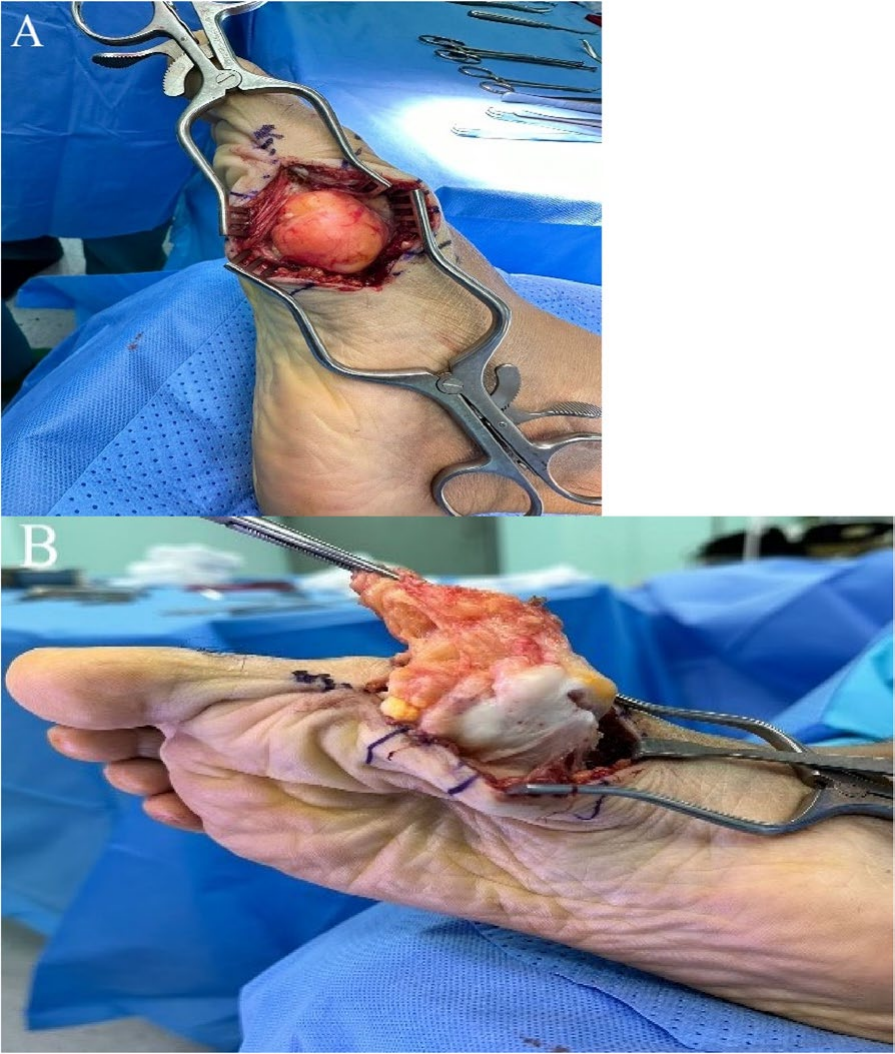
**A**.** B**. Intraoperative view following fascial dissection showing a large mass composed of fibrous and chondral tissues

**Fig. 6:**
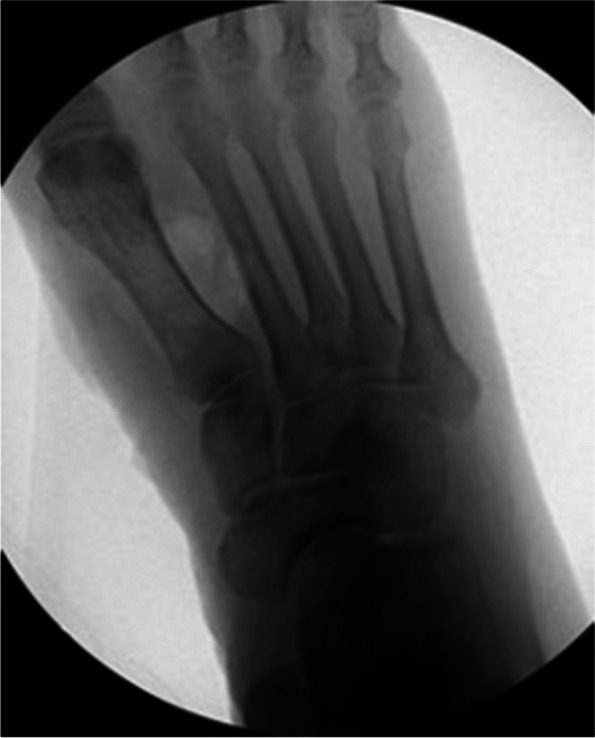
Intraoperative radiograph of the right foot

 The operation was performed under general anaesthesia, with the patient in the supine position. A 350 mmHg torniquet was applied, and a medial incision was made (Fig. 4). Following dissection of the fascia, the mass was observed to be large, composed of fibrous and chondral tissues, and reaching between the first and third metatarsal bones (Fig. 5). Total excision was performed, and triple washout with hydrogen peroxide, iodine, and saline was performed. The mass was sent to histopathology for confirmation of the diagnosis. The wound was sutured in layers with vicryl 1 and vicryl 0 the vicryl 2.0, the skin was closed with monocryl 4.0., and pressure dressing was applied. The patient was in stable condition and had intact vascularity. The excisional biopsy measured 5.5 × 3.5 × 3.0 cm. The lesion was observed to contain mature fatty tissue with areas of fibrous and cartilaginous tissue and was negative for malignancy. The intraoperative radiograph is shown post-excision (Fig. 6).

**Fig. 7:**
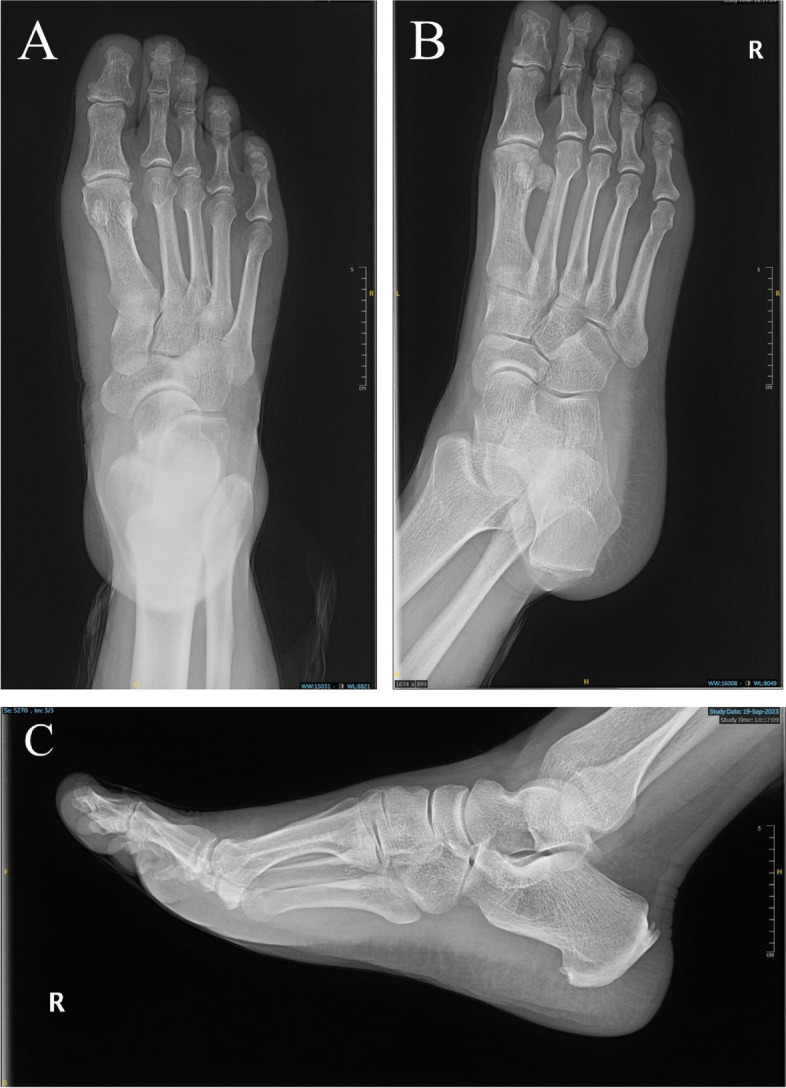
Postoperative radiographs of the right foot **A**. AP view **B**. Oblique view. **C** Lateral view

Postoperatively, the patient had intact neurovascularity and experienced no complications. He was discharged home in stable condition with appropriate analgesia and a course of prophylactic antibiotic. He was advised for toe touch ambulation and further follow-up in the clinic, and had been on an ankle brace for two weeks. After the two weeks, the wound demonstrated appropriate healing, and sutures were removed. There had been no signs of infection or wound dehiscence. The patient commenced gradual range of motion two weeks postoperatively and had reached full range within one week. He returned to his normal activities four weeks following the excision. Radiographic imaging on the 8-month follow-up are shown (Fig. 7)

## Discussion

Osteochondrolipoma is a benign lesion considered as a histological variant of lipoma. Few cases have been reported in the literature on this lesion in the mandible, chest wall, hand, scapula and ischium, indicating a limited number of occurrences [4–7]. According to Kitazawa et al., the maxillofacial region was reported as the most frequent location, with infrequent occurrence in the lower limb. The average patient age was also observed to be 57.4 years, with no clear gender predominance [7]. To date, we believe this represents the 18th case of osteochondrolipoma reported in the literature. According to our review of the literature with the keywords “osteochondrolipoma” and “ossifying chondrolipoma”, this is the first case reported to affect the foot.

Although it remains unclear, multiple theories have been suggested for the pathogenesis of osteochondrolipomas. One theory suggests that the different components independently arise from multipotent mesenchymal cells, while another suggests it indicates a metaplastic process in a previously existing chondrolipoma or lipoma, and some suggest repetitive trauma to cause secondary ossification [7–9]. Furthermore, the diagnosis of osteochondrolipomas depends on plain radiographs, computerized tomography (CT), MRI, and histopathology. MRI is regarded as the optimal imaging modality in this condition. To avoid misdiagnosis, a histopathological evaluation of the whole tumor, instead of an incisional biopsy, is the preferred method for diagnosis.

A variety of observations regarding its presentation have been made in the literature. Some case reports described it as firm, mobile, and nonadherent to muscle or bone, while others found it firmly attached. Furthermore, symptom presentation may also differ. In certain instances, and as opposed to scapular lesions, patients with osteochondrolipomas of the hand may present with complaints of pain and numbness [1, 6, 8]. The patient in our case experienced progressively worsening pain associated with swelling, eventually leading to a significant impact on their daily activities. Myositis ossificans, calcified or ossified tumours, hemangiomas, calcified bursae, and well-differentiated liposarcomas are all differentials of this condition [10].

In contrast to this condition, and on MRI, benign soft tissue tumors show uniform high intensity on T1, with low signal intensity appreciated on T2 weighted images [11]. On the other hand, myxoid liposarcomas show a well-defined and multilobular mass within the subcutaneous tissue, with T1-weighted images demonstrating low signal intensity with foci of linear high signal intensity, and heterogeneously high signal intensity on T2-weighted images [12].

In this rare location of osteochondrolipoma, we excised the lesion through the medial approach. We tried to avoid the plantar approach to avoid violating the plantar fascia or transecting nerve endings that may lead chronic pain with weight bearing. Chronic incisional pain was seen in 7.1% of patients who were treated with the plantar approach, and 5.1% showed a hypertrophied scar. Delayed wound healing was also noticed, and another study reported a 27% incidence of chronic incisional pain with planter approach [13].

In this study, we present the first osteochondrolipoma that presented in the foot. Our investigations included x-rays, MRI’s and our treatment modality of choice was through with surgical excision by using a medial approach to avoid complications of the plantar approach. The patient showed no chronic incisional pain, hypertrophic scar, or cyst formation.

## Conclusion

Osteochodrolipoma is a benign lesion that can affect the foot leading to decreased functionality of the foot due to the pain and swelling. Surgical excision is the recommended approach for this lesion, providing both symptomatic relief and confirmation of the diagnosis through histopathological examination.

## Data Availability

No datasets were generated or analysed during the current study.

## Abbreviations

CT: Computerized tomography
MRI: Magnetic resonance imaging
AP: Anteroposterior

## References

[1] Murphey MD, Carroll JF, Flemming DJ, Pope TL, Gannon FH, Kransdorf MJ (2004). From the archives of the AFIP: benign musculoskeletal lipomatous lesions. Radiographics.

[2] İncekara F (2020). Is the treatment for osteochondrolipomas and lipomas the same? Case report and review of the literature on osteochondrolipoma of chest wall. Turkish J Thorac Cardiovasc Surg.

[3] Rau T, Soeder S, Olk A, Aigner T (2006). Parosteal lipoma of the thigh with cartilaginous and osseous differentiation: an osteochondrolipoma. Annals Diagn Pathol.

[4] Nishio J, Ideta S, Iwasaki H (2013). Scapular osteochondrolipoma: imaging features with pathological correlation. Oncol Lett.

[5] Zhu J, Li Y, Fan M, He X, Wang L. Osteochondrolipoma: a lipoma with cartilaginous and osseous differentiation of the ischium. U.S. National Library of Medicine; 2018. https://www.ncbi.nlm.nih.gov/pmc/articles/PMC6962959/. Accessed 26 Nov 2023.

[6] Van Demark Jr RE, Fiegen T, Hayes M, Hayes M, Sunassee A, Helsper E. Osteochondrolipoma of the hand. J Hand Surg. 2022;47(9):904–e1. 10.1016/j.jhsa.2021.05.024 34312026

[7] Kitazawa T, Shiba M. Osteochondrolipoma of the mandible. U.S. National Library of Medicine; 2017. https://www.ncbi.nlm.nih.gov/pmc/articles/PMC5712529/. Accessed 26 Nov 2023.

[8] Choi Y-J, Kang J-H, Kang G-H, Choi S-J (2015). Osteochondrolipoma presenting as a popliteal cyst. Clin Orthop Surg.

[9] Taha MF, Hedayati V (2010). Isolation, identification and multipotential differentiation of mouse adipose tissue-derived stem cells. Tissue Cell.

[10] Heffernan EJ, Lefaivre K, Munk PL, Nielsen TO, Masri BA (2008). Ossifying lipoma of the thigh. Br J Radiol.

[11] Derin AT, Yaprak NJ (2017). Lipomas. Review and evaluation of the literature. Clin Surg.

[12] Kure S, Peng WX, Kudo M, Matsubara M, Tsunoda T, Naito Z (2014). A rare case of myxoid liposarcoma of the adult foot diagnosed using fine needle aspiration cytology (FNAC). Pathology and Laboratory Medicine International.

[13] Masaragian HJ, Perin F, Rega L, Ameriso N, Mizdraji L, Coria H, Cicarella S (2021). Minimally invasive neurectomy for Morton’s neuroma with Interdigital approach. Long term results. Foot.

